# Prevalence of Depression in Patients With Post-Acute Coronary Syndrome and the Role of Cardiac Rehabilitation in Reducing the Risk of Depression: A Systematic Review

**DOI:** 10.7759/cureus.20851

**Published:** 2021-12-31

**Authors:** Zahid Khan, Khalid Musa, Mohammed Abumedian, Mildred Ibekwe

**Affiliations:** 1 Cardiology, Royal Free Hospital, London, GBR; 2 Internal Medicine, Barking, Havering and Redbridge University Hospitals NHS Trust, London, GBR; 3 Geriatrics, Barking, Havering and Redbridge University Hospitals NHS Trust, London, GBR; 4 Cardiology and Internal Medicine, Barking, Havering and Redbridge University Hospitals NHS Trust, London, GBR

**Keywords:** nine-item patient health questionnaire (phq-9), systematic review and meta-analysis, american heart association, cardiac rehabilitation, beck's depression inventory questionnaire, hospital anxiety and depression scale questionnaire, acute myocardial infarction, acute coronary syndrome

## Abstract

Patients with acute myocardial infarction (AMI) or ischaemic heart disease are at risk of developing anxiety and depression. This systematic review aims to identify the various risk factors and the role of cardiac rehabilitation in reducing the risk of depression in patients after AMI. In this review, we included data on the prevalence of depression in patients post-AMI for the years 2016-2017 from a cardiac rehabilitation unit at Morriston Hospital, Swansea, a primary coronary angioplasty centre. Results from our meta-analysis were compared with the findings of previous studies. Our data showed the prevalence of depression to be 14% pre-cardiac rehabilitation and 3% post-cardiac rehabilitation. A meta-analysis of seven studies showed the prevalence to be approximately 20-35% depending on the type of questionnaire or screening method used. Gender, marital status, age, and sedentary lifestyle were found to be risk factors for depression post-acute coronary syndrome (ACS). Females, patients aged >65 years, and those who were single, lived alone, or widowed were at a higher risk of depression, and patients with sedentary lifestyles were more likely to have post-ACS depression. Depression in patients post-myocardial infarction was also associated with increased mortality and morbidity risk as well as higher hospital re-admission and future cardiac events.

The meta-analysis showed significant publication bias, studies with negative results were less likely to be published, and the study data were heterogeneous. The pooled estimate for depression estimated using the random-effects model was 1.78 (95% confidence interval = 1.58-2.01).

## Introduction and background

Depression is common in patients with ischaemic heart disease (IHD). The number of patients admitted with acute coronary syndrome (ACS) in the United States is approximately 2.4 million every year. Post-ACS depression is an independent risk factor for future cardiac events and mortality, affecting more women than men [1,2]. The annual mortality in Europe from ACS and IHD is 1.8 million. Approximately 785,000 new and 470,000 recurrent myocardial infarctions (MIs) are reported every year in the United States [3]. In the United Kingdom, approximately 110,000 men and 65,000 women suffer from acute myocardial infarction (AMI). On average, someone has an AMI event every three minutes in the United Kingdom [4]. A total of 2.3 million people in the United Kingdom live with coronary artery disease (CAD) due to improved treatment and survival rates. Research has shown a higher prevalence of depression and anxiety in patients post-ACS [5].

Lespérance et al. (1999) reported that 47% of female patients post-ACS had symptoms of depression during hospital admission [1]. Additionally, the American Heart Association (AHA) Populations 2011 Update reported approximately 7.5 million women living with depression [6]. Women are not only twice more likely to suffer from depression compared to men they also suffer from significant depressive symptoms [7,8]. Patients with depression are less likely to adhere to cardiac rehabilitation advice and are at an increased risk of death during the initial few months of ACS [9]. Haq Nawaz and Shehzad (2016) reported the prevalence of depression to be 19.4% in patients following AMI, with women being more likely to suffer from depression [8,10]. Anxiety and depression are more prevalent in patients with following AMI than in the general population and remain underdiagnosed and undertreated [11]. The AHA recommends routinely screening patients with ACS for depression due to higher risk; however, depression as a risk factor for ACS was reported in 2014 [12]. Meneghetti et al. (2017) reported that 15-20% of hospital inpatients met the criteria for major depression and another 25-65% of patients reported at least one symptom of depression compared to the lifetime prevalence of 10% in the general population [9,13].

Clinical depression is an independent risk factor for IHD, and one possible explanation for this is the enhanced platelet activity in these patients [14]. The Johns Hopkins Precursors Study utilised a prospective design and was based on 1,190 male medical students enrolled between 1948 and 1964 who reported higher mortality and morbidity in patients post-MI with clinical depression. Studies have shown that patients with simultaneous generalised anxiety disorder (GAD) and depressive disorder exhibit a higher risk of cardiac death, suggesting that GAD and depressive disorders may act synergistically to affect CAD [14]. On the other hand, it has been estimated that the total annual cost of depression in England in 2007 was £7.5 billion, and the resultant lower productivity accounted for a further cost of £1.7- £2.8 billion while the cost for anxiety was £1.2 billion in 2007 alone [15]. Most studies on the prevalence and incidence of depression post-MI do not provide sufficient data. This important aspect of the rehabilitation program has been ignored. Sunamura et al. (2018) reported 46% lower mortality in patients who completed cardiac rehabilitation compared to those who did not [14,16].

## Review

Literature search

A literature search was carried out using Cochrane Library, Embase, Medline, ScienceDirect, PscyArticles, PsycINFO, and PubMed databases. Only articles published after 2000 were included in this systematic review. Previous studies have used various questionnaires including the Hospital Anxiety and Depression Scale (HADS), Patient Safety Questionnaire, and Beck Depression Inventory (BDI) questionnaire. There was a lack of uniformity and considerable variation in the choice of questionnaires used in these studies, with different studies reporting one or the other questionnaire to be superior at detecting depression among patients. Moreover, other scales such as the Cardiac Depression Scale, Cardiac Depression Visual Analogue Scale, Hamilton Depression Scale, Patient Health Questionnaire (PHQ)-9, and PHQ-2 have also been utilised by several studies. This lack of uniformity may over or underestimate the prevalence of depression in AMI patients. Most studies failed to provide any information regarding the risk of depression or anxiety in these patients prior to AMI.

**Figure 1:**
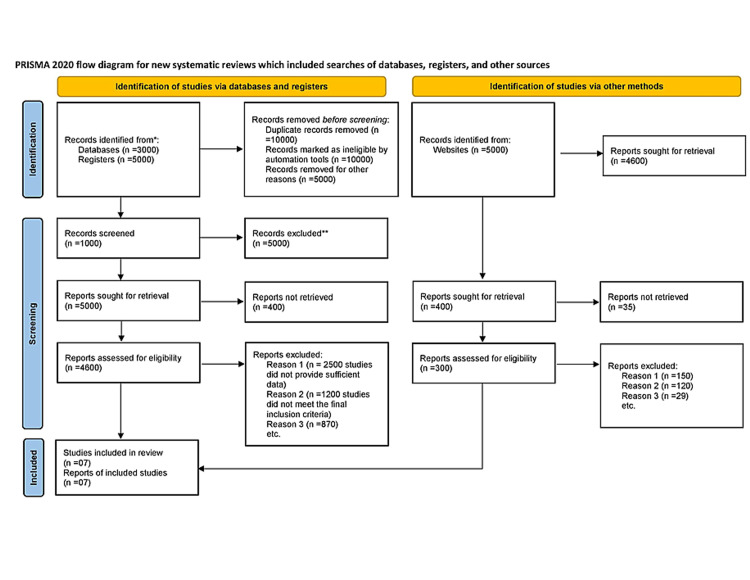
PRISMA flow diagram for new systematic reviews which included searches of databases and other sources. PRISMA: Preferred Reporting Items for Systematic Reviews and Meta-Analyses

The following keywords were used for the literature search: acute coronary syndrome, depression in ACS, the prevalence of depression in ACS, ACS and depression, ACS and anxiety, acute myocardial infarction (AMI) and anxiety, acute myocardial infarction and depression, acute myocardial infarction and anxiety, ischaemic heart disease and anxiety, ischaemic heart disease and depression, HADS questionnaire, PHQ questionnaire, and BDI questionnaire. Over 40,000 articles met the initial search criteria, but the list was further narrowed down after reviewing the title and abstract. Less than 50 articles met the inclusion criteria after reviewing the abstract. Finally, 40 studies were excluded as they did not provide enough data to meet the inclusion criteria. We were not able to access three studies. The literature search criteria and methodology were agreed upon by two independent researchers who also agreed with the selection of studies. The Preferred Reporting Items for Systematic Reviews and Meta-Analyses and the PICO flow diagram used for the literature search and methodology are presented in Figure 1.

Methodology

A meta-analysis was performed on the selected studies as well as our data from the cardiac rehabilitation centre at Morriston Hospital. Data were retrospectively collected for patients attending the cardiac rehabilitation unit at Morriston Hospital from 2016 to 2017 post-AMI. The PHQ, BDI and HADS questionnaires were commonly used to collect data in the selected studies, and the HADS questionnaire was used for data collection in Morriston Hospital. Studies were included in this review irrespective of the type of questionnaire used if sufficient information on patient demographics and the depression status pre and post-AMI was provided.

Inclusion criteria

Studies were eligible for inclusion in the systematic review if the following conditions were met: (1) study participants were >18 years of age; provided sufficient data for patient demographics and pre-AMI depression status; (3) provided sufficient patient demographic data irrespective of other data regarding patient education and comorbidities; and (4) participants were treated for either acute non-ST-elevated myocardial infarction (NSTEMI), ST-elevated myocardial infarction (STEMI), or unstable angina (UA).

Exclusion criteria

Studies were excluded from this systematic review if the following conditions were met: (1) study participants were <18 years of age; (2) data on patients’ demographics and depression were not provided; (3) participants were treated for a cardiovascular condition other than ACS; (4) participants were treated for ACS and post-ACS depression but failed to provide information on patients’ pre-ACS depression status; and (5) conducted before 2000.

In total, 10 studies met the inclusion criteria and were included in the review; however, only seven studies were included in the meta-analysis. Only a few studies provided sufficient data about patient demographics and any attributable risks such as diabetes, hypertension, hypercholesterolaemia, and level of patient education. Most reviews assessed the prognosis in patients with ACS, the validity of questionnaires, the treatment effectiveness in patients with depression after ACS [17,18]. SPSS (IBM Corp., Armonk, NY, USA) software was used for data analysis, and the meta-analysis was performed using the “R” statistical program to calculate the odds ratio (OR) for random-effect models.

Quality assessment

The methodological quality of eligible studies was assessed using the checklist of Prevalence Study Quality (QUADAS checklist) which is widely used to evaluate the methodological quality of studies. It consists of 11 items with response options of “Yes,” “No,” or “Unclear,” and the final score ranges from 0 to 11. If the response for an item is “Yes,” it is scored “1,” otherwise it is scored “0.” Therefore, the total score for this instrument ranges from 0 to 11, and studies are categorised as low quality, moderate quality, and high quality with a total score of 0-3 points, 4-7 points, and 8-11 points, respectively [19].

Results

A total of 380 patients joined the cardiac rehabilitation unit at Morriston Hospital in 2016 and 2017. Overall, 324 patients completed the cardiac rehabilitation program (CRP). The HADS questionnaire was used to screen patients for depression at the beginning and on completion of the CRP. Overall, 51 patients who joined the CRP had a HADS score of more than 8 upon joining the program. From 2016 to 2017, 43 patients from the rehab program completed the HADS questionnaire on joining and completing the program. In total, eight female and thirty-five male participants completed both questionnaires. The minimum score recorded from the enrolled patients was 9 and the maximum score was 16 on joining the program. In total, 16 patients (twelve male and four female) had a HADS score of 9 and 27 patients (nine female and eighteen male) had a HADS score of greater than 10. On completion of the rehab program, only eight patients (two female and six male) had a HADS score >8. A HADS score of >8 was used to confirm the presence of depression symptoms in previous studies. The chi-square value for depression based on gender was 0.265 with one degree of freedom (χ^2^(1, N = 43) = 0.265; p > 0.05). Of the 43 patients, 29 were married, four were in a long-term relationship, and 10 were living alone. Of the 10 patients living alone, four were separated, four were widowed, and two were single. Our findings showed a decline in the prevalence of depression among patients on the completion of the CRP. The findings from this study are consistent with previous studies reporting that female patients are more likely to suffer from depression after a heart attack. The prevalence of depression decreased from 14% to 3% on completion of the CRP.

**Figure 2:**
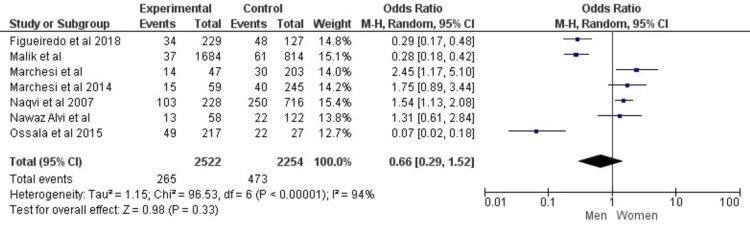
Risk of depression based on Gender Ossola et al. [20], Marchesi et al. [21], Mallik et al. [22], Naqvi et al. [23], Haq Nawaz and Shehzad [8], Ossola et al. [24], Figueiredo et al. [25]. CI: confidence interval

Most studies identified in the literature search did not provide sufficient data to calculate OR and confidence interval (CI) for depression; hence, these studies were excluded from the meta-analysis. The funnel plot for meta-analysis suggested the presence of publication bias as the plot asymmetry showed that negative studies are unlikely to be published. The following seven studies included in the meta-analysis: Ossola et al. [20], Marchesi et al. [21], Mallik et al. [22], Naqvi et al. [23], Haq Nawaz and Shehzad [8], Ossola et al. [24], Figueiredo et al. [25]. Both the fixed-effect and random-effect model values were calculated; however, as the data were heterogeneous, only the random-effect model was chosen. A previous meta-analysis demonstrated that patients with depression were 1.7822 times more likely to experience ACS (95% CI = 1.5802; 2.0101; p < 0.0001). The findings suggested that patients, particularly females, were more likely to suffer from depression post-MI. The Tau^2 ^heterogeneity test value for gender-associated risk of depression was 1.15, degree of freedom (df) = 6, I^2 ^= 94% (P < 0.00001); OR for men:women was 0.66 (95% CI = 0.29-1.52) based on the random-effect model, as shown in Figure 2.

Ossola et al. (2015) reported OR to be 1.69 with 95% CI (1.3211; 2.1618), fixed weightage of 2.0%, and random weightage of 14.6%. The maximum weightage effect was reported by Naqvi et al. (2007) with an OR of 1.6400, 95% CI (1.5813; 1.7009), and random weightage 36.1%. The highest weightage among these studies was reported by Naqvi et al. (2007) with a point estimate of 1.66, 95% CI (1.58; 1.70), fixed weightage of 89.5%, and random weightage of 36.1%. The smallest weightage was reported by Marchesi et al. (2014) with a point estimate of 2.27, and 95% CI (0.65; 7.90), fixed weightage of 0.1%, and random weightage of 0.9%. The CI was large in these studies which may explain the observed dispersion among the studies. The weightage for each individual study provides information regarding the effects of each study on the final weightage of the meta-analysis [26].

The test for heterogeneity I^2 ^in this study was highly significant with a Q(6) of 12.37 and p of 0.0543; hence, the random-effect model was selected due to the heterogeneity of the data. The I^2 ^value for heterogeneity of the meta-analysis was 51%, confirming a moderate level of heterogeneity. Women were more likely to suffer from depression based on the findings of this meta-analysis, and the point estimate for the random-effect model was 1.7822, 95% CI (1.5802; 2.0101) (P < 0.0001). A significant intraobserver variation was seen among studies and the CI was quite wide in a few studies. Tau^2^ was 0.0101 and I^2^ was >51%, confirming a moderate level of variation.

**Figure 3:**
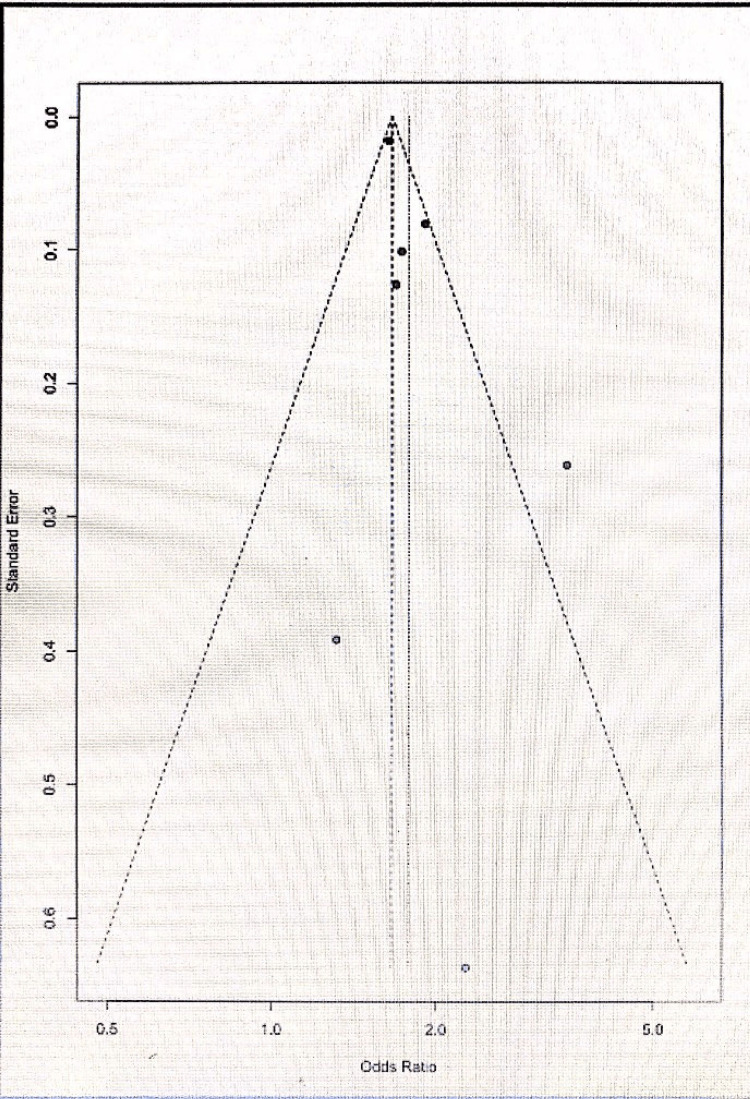
Funnel plot for the meta-analysis.

**Figure 4:**
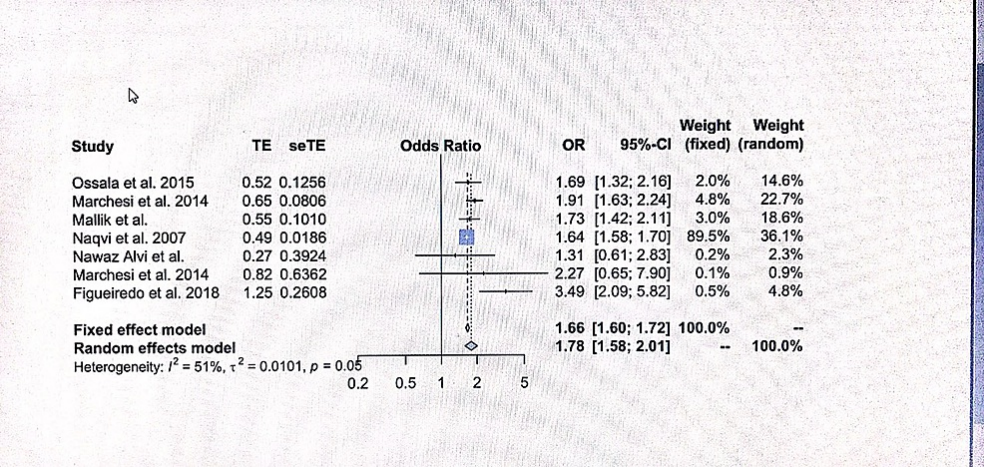
Meta-analysis forest plot. Ossola et al. [20], Marchesi et al. [21], Mallik et al. [22], Naqvi et al [23], Haq Nawaz and Shehzad [8], Ossola et al. [24], Figueiredo et al. [25]. OR: odds ratio; CI: confidence interval

**Figure 5:**
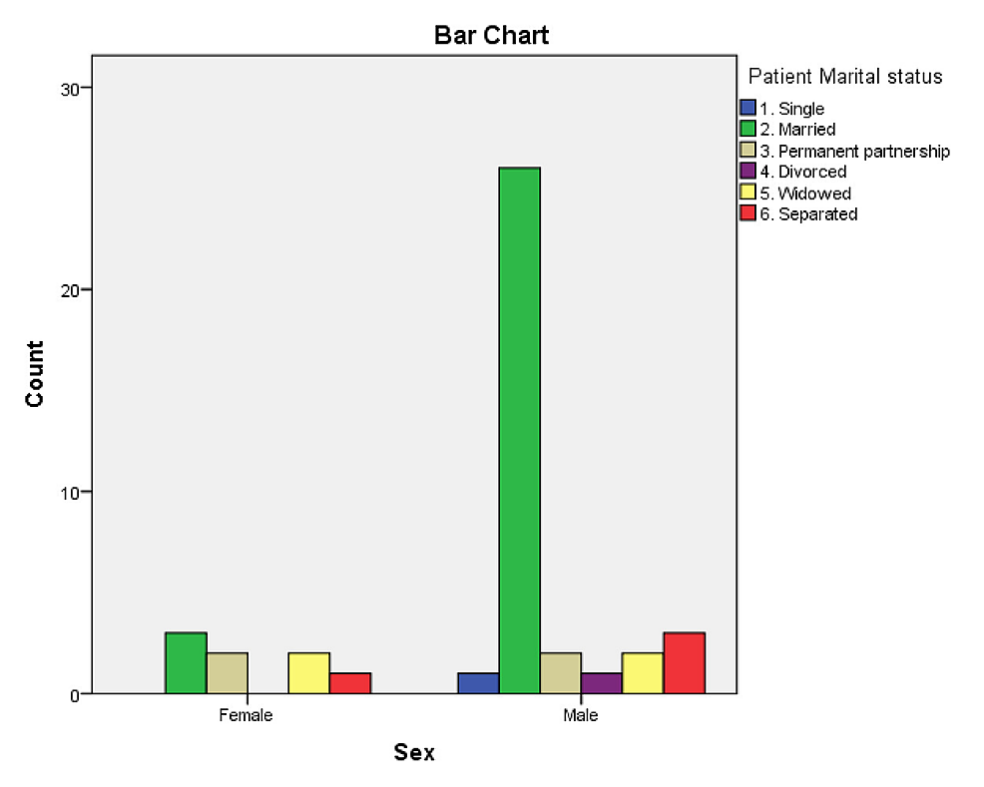
Marital status data for cardiac rehabilitation patients of Morriston Hospital.

It is also worth mentioning that several studies with a significant number of participants could not be included in the meta-analysis because very limited data were provided regarding patient characteristics. The prevalence of depression varies in patients post-ACS depending on the method of assessment. A Danish study not included in the meta-analysis based on the data of 97,793 Danish patients from the Danish ACS registry reported that 10,106 men (total number of men = 60,361) and 9,414 women (total number of women = 37,432) had symptoms of depression post-ACS. The overall prevalence of depression in the study was 32%, with a higher prevalence among women. The study results are shown in Figures 2-5 and Table 1. A major problem with this study was that information was retrieved from the Danish registry, the severity or diagnosis of depression was based on coding, and a significant proportion of these patients were on antidepressants for other reasons that could have resulted in the overestimation of depression and underestimation of less severe depression. In addition, the study had a significant number of patients for whom the type of myocardial event was not clear. Another factor to exclude this study from the meta-analysis was that the authors analyzed depression as a time-dependent variable using the landmark method, with follow-up at pre-specified time points. If an individual died prior to the landmark point, they were not included in the analysis which could have resulted in bias.

**Figure 6:**
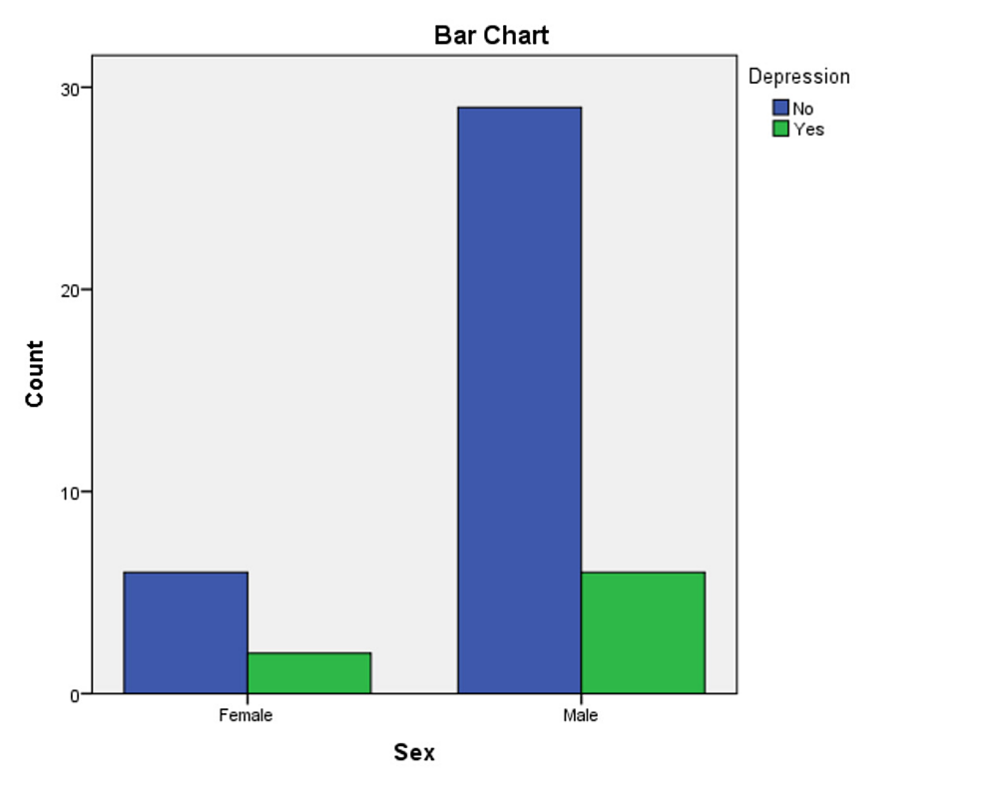
Depression data for cardiac rehabilitation patients of Morriston Hospital.

**Table 1:** Chi-square analysis for cardiac rehabilitation centre phase 02. ^a^30 cells (100.0%) have an expected count of less than 5. The minimum expected count is 0.19. ^b^Based on 10,000 sampled tables with starting seed of 2,000,000. df: degree of freedom; CI: confidence interval

Chi-square test						
Test	Value	df	Asymptotic significance (two-sided)	Monte Carlo Significance (two-sided)		
				P-value	95% CI	
					Lower bound	Upper bound
Pearson chi-square	10.202^a^	14	.747	0.821^b^	0.813	0.828
Likelihood ratio	12.040	14	.603	0.800^b^	0.792	0.808
Fisher’s exact test	11.225			0.785^b^	0.777	0.793
Number of valid cases	43					

Discussion

The findings of this meta-analysis show that depression is common in patients suffering from AMI, with women at a higher risk compared to men. The OR for the random-effect heterogeneity model was 1.78, 95% CI (1.58-2.01), I^2^ 51%. The Tau^2 ^for gender-based depression was 1.15, Q6, I^2^ 94% (P = 0.00005). The maximum possible score was 21; a score of 0-7 was considered normal, a score of 8-10 showed moderate depression, and a score greater than 10 showed severe depression. A previous meta-analysis showed that by using a cut-off score of 8 for depression, HADS could detect major depression with 82% sensitivity and 74% specificity [27].

Research has shown that smoking, drinking, lack of exercise, poor medical compliance, and physiological changes in the body such as increased sympathetic nervous system activity are associated with increased risk of depression post-ACS [22].

Figueiredo et al. (2017) reported gender, sedentary lifestyle, age of <60 years, and previous history of major depression were associated with increased risk of depression post-AMI. The higher prevalence of depression (23%) in patients aged >60 post-MI was statistically insignificant and was more common in women. Depression was positively associated with the marital status of patients and was more common in patients who were single or widowed. There was a positive association between depression and socioeconomic status and years of schooling, and prevalence was high among patients with poor socioeconomic backgrounds and in those with less than four years of schooling. A sedentary lifestyle was associated with an increased risk of depression, although it was not associated with smoking, hypertension, or diabetes. Patients who had previous AMI were less likely to suffer from depression compared to patients presenting with AMI for the first time. A sedentary lifestyle was found to be associated with depression, and sedentary female patients were less likely to suffer from depression compared to male patients [27].

The reason for the increased risk of depression in women post-AMI is not clear, although one study reported an association between neuroticism and female gender and depression [28]. Frazier et al. (2012) reported that women were twice more likely to suffer from somatic symptoms compared to men. This meta-analysis confirmed the findings of previous studies that women are at a higher risk of suffering from depression post-AMI. Younger women with depression post-AMI tend to have a worse prognosis compared to men of similar age, which can be partially attributed to coexisting conditions, clinical characteristics, and early management [29].

There is significant publication bias in the published studies overestimating the prevalence of depression in patients with ACS [30]. Aetiological studies have shown the presence of publication bias in the adjusted results, and studies with significant unadjusted results were likely to report adjusted effects. Moreover, the pooled estimate was likely to be smaller if the adjusted effect was provided by all studies. This may have resulted in associating depression with poor prognosis post-AMI [31].

A Danish study on 97,793 patients hospitalised between 2001 and 2009 for ACS reported the presence of depression in 20% of patients within two years post-ACS, with an adjusted rate ratio of 1.28, 95% CI (1.25; 1.30). Overall, 7,393 patients were new cases of depression and 12,127 had previous episodes of depression. In total, 54,737 and 43,056 patients were living with their partners and alone, respectively. Overall, 43,951 patients had more than three comorbidities, 45,441 had one or two comorbidities, and 8,401 patients had no comorbidities. This study also confirmed the findings of previous studies with high prevalence and incidence of depression in patients living alone, those with basic education, and those with prior depression or other comorbidities. The reported prevalence in this study is smaller compared to a previous study that reported it to be 30-40%. The hazard ratio (HR) for ACS patients with previous depression was 1.33, 95% CI (1.29; 1.38), for recurrent depression was 1.62, 95% CI (1.57; 1.67), and for new-onset depression was 1.66, 95% CI (1.60; 1.72). The study reported higher mortality rates than patients with no depression. There was no difference in mortality rates based on the timing of onset of depression within 30 days of ACS or later. Studies have linked depression to poor prognosis post-ACS; however, Zuidersma et al. (2011) reported a lack of evidence to support this idea.

The VIRGO study, a multinational and multicentre study based on 3,572 patient’s data with AMI, reported 48% of women to have symptoms of depression in their lifetime, and 39% and 22% of women and men, respectively, reported symptoms of depression at the time of admission with AMI, with adjusted OR of 1.64; 95% CI (1.36; 1.98) [27]. Overall, 51% of the study participants were married, 95% of the participants attended higher school or above and 62% of participants were part or full time employed. The OR for depression for females was 2.28 compared to 1.78 in our study and the OR was 1.64, 95% CI (1.36; 1.98), after adjusting for various demographic factors such as socioeconomic status, health, and education. Depression was more prevalent in younger women compared to men prior to ACS and had 60% higher odds compared to men after adjusting for various demographics [30].

Meneghetti et al. (2018) reported the prevalence of depression and anxiety to be 26.4 and 48.4%, respectively, in the cross-sectional study based on 91 patients. Only 19 patients (20% of the study population) had HADS scores indicative of both anxiety and depression in this study group. Amin et al. (2006) reported the prevalence of depression to be 17.6% in their cross-sectional study based on 1,181 ACS patients with a mean age of 61.5 ± 12.9 years. The majority of this patient group was males and Caucasians with a total number of 208 patients, out of which 57 had recognised symptoms of depression and 157 had unrecognised symptoms of depression. This study also highlighted that approximately 25% of patients had documentation of moderate-to-severe depression in their notes prior to discharge post-ACS. Lespérance et al. reported in their study that 8% of inpatients with post-ACS depression had psychiatric consultation and only 1% had antidepressant medications. Smokers and ex-smokers were at a higher risk of suffering from post-ACS depression in the study, and the HAD-A score was 7.89 and the HAD-D score was 5.27 for the study population. Patients with a combination of GAD and depression post-ACS were more likely to have a fatal outcome secondary to cardiac problems reported in another study [30].

The Enhancing Recovery in CHD Patients (ENRICHD) study reported a prevalence of 20% based on an examination of 9,279 patients; however, a major problem with the ENRICHD study was that it included patients with low socioeconomic status and major depression only. The prevalence of depression was noted to be 10-47% for patients in the 17 studies which used validated questionnaires for screening patients for depression. Nine studies included in this systematic review used the BDI questionnaire score of ≥10 to indicate mild-to-moderate depression, the reported prevalence was 20-37%, and the weighted prevalence was 31.1%, 95% CI (29.2; 33.3). Four studies used the HADS questionnaire and the prevalence was 11-17%, with a weighted prevalence of 15.5%, 95% CI (13.2; 18.0). This systematic review showed similar depression prevalence in studies where a similar assessment method was used; however, significant differences were noted in the weighted prevalence of depression in studies where different questionnaires or assessment methods were used [30-32].

Cardiac rehabilitation is offered to patients for various cardiovascular conditions such as heart failure, MI, and congenital heart disease. There are four stages of cardiac rehabilitation, namely, the acute phase, the sub-acute phase, intensive outpatient therapy, and independent ongoing conditioning [31]. Phase 1 of cardiac rehabilitation starts during hospital admission. An acute physical therapist works closely with doctors, nurses, and other staff involved in patient health care. During phase 2, the patient attends the outpatient clinic which lasts for approximately three to six weeks and focuses on response to activity and exercise. Phase 3 involves more independent cardiac rehabilitation, and the patient monitors their heart rate, response to exercise, and exercise tolerance or capacity. Moreover, physiotherapists help to increase their exercise tolerance. In phase 4 of cardiac rehabilitation, patients continue to focus on their cardiac response and work on their independence and ongoing conditioning of the heart and physical health. Studies have shown that patients who join cardiac rehabilitation after angioplasty have a higher survival rate [16]. This study also reported 39% lower mortality in post-ACS patients who joined cardiac rehabilitation which was 46% lower in patients who completed CRP. The 10-year mortality for the first group was 14.7% versus 23.5%, with an adjusted HR of 0.61 [95% CI (0.46; 0.81)]. The 10-year mortality for the latter group was 13.6% versus 18.9%, with an adjusted HR of 0.54 [95% CI (0.42; 0.70)]. Cardiac rehabilitation not only improves the physical and psychological health of the patients but also reduces hospital re-admission rates [33,34]. This meta-analysis showed that patients with depression or who develop depression post-AMI are at a higher mortality risk, as confirmed by most studies, although few studies have reported contradictory results [35].

Study limitations

The findings of this study are limited due to the lack of uniformity in the types of questionnaires used to collect data in previous studies which could have affected the results. In addition, most studies did not provide enough data for patient demographics and depression.

## Conclusions

This systematic review shows that depression is common in patients post-ACS and remains undertreated which can result in higher morbidity and mortality and lead to increased hospital re-admission. Therefore, patients should be encouraged and offered cardiac rehabilitation to help them enhance their performance and improve exercise tolerance. Although post-ACS care has improved for patients, there is plenty of work to do. Patients should be advised on lifestyle modification to reduce future risk of cardiac events, and antidepressants may have a role if they show signs of depression. The various scales and questionnaires currently used are subjective and report higher or lower prevalence at times. By providing cardiac rehabilitation and timely treatment to patients showing symptoms of depression, their future cardiac event risk can be reduced. Moreover, their hospital re-admissions can also be prevented which could decrease financial costs to hospitals and enable patients to lead a more independent life and adopt a healthier lifestyle. Although there are various screening methods to screen patients for depression after ACS, a single method used universally would provide more realistic data regarding the prevalence of depression; however, each assessment or screening method has its own limitations.

As the prevalence varies considerably from 15% to 40%, future studies involving larger datasets to assess the actual prevalence of depression in AMI patients would be useful. In addition, any future research should involve patients from different ethnic backgrounds to assess the true prevalence across various ethnic groups to better determine the risk factors. In addition, due to the lack of a universal scale to screen patients for symptoms of depression and anxiety, the prevalence tends to differ between studies, and a universal screening tool would be valuable to measure the true prevalence of depression in patients after cardiac events. Finally, we recommend that patients should be assessed for depression after suffering from MI during admission and at the one-month post-discharge follow-up.

## Appendices

**Table 2:** QUADAS checklist. QUADAS: Quality Assessment of Diagnostic Accuracy Studies

Item	Yes	No	Unclear
1. Was the spectrum of patients’ representative of the patients who will receive the test in practice?	1	0	0
2. Were selection criteria clearly described?	1	0	0
3. Is the reference standard likely to correctly classify the target condition?	1	0	0
4. Is the time period between reference standard and index test short enough to be reasonably sure that the target condition did not change between the two tests?	1	0	0
5. Did the whole sample, or a random selection of the sample, receive verification using a reference standard of diagnosis?	1	0	0
6. Did patients receive the same reference standard regardless of the index test result?	1	0	0
7. Was the reference standard independent of the index test (i.e., the index test did not form part of the reference standard)?	1	0	0
8a. Was the execution of the index test described in sufficient detail to permit replication of the test?	1	0	0
8b. Was the execution of the reference standard described in sufficient detail to permit its replication?	1	0	0
9a. Were the index test results interpreted without knowledge of the results of the reference standard?	1	0	0
9b. Were the reference standard results interpreted without knowledge of the results of the index test?	1	0	0
10. Were the same clinical data available when test results were interpreted as would be available when the test is used in practice?	1	0	0
11. Were uninterpretable/ intermediate test results reported?	1	0	0
12. Were withdrawals from the study explained?	1	0	0
Total score	11	0	0
